# Hydroxychloroquine and risk of cancer in patients with primary Sjögren syndrome: propensity score matched landmark analysis

**DOI:** 10.18632/oncotarget.19057

**Published:** 2017-07-06

**Authors:** Yao-Fan Fang, Yen-Fu Chen, Ting-Ting Chung, Lai-Chu See, Kuang-Hui Yu, Shue-Fen Luo, Chang-Fu Kuo, Jenn-Haung Lai

**Affiliations:** ^1^ Division of Rheumatology, Allergy and Immunology, Chang Gung Memorial Hospital, Taoyuan, Taiwan; ^2^ Biostatistics Core Laboratory, Molecular Medicine Research Centre, Chang Gung University, Taoyuan, Taiwan; ^3^ Division of Rheumatology, Orthopaedics and Dermatology, School of Medicine, University of Nottingham, Nottingham, England; ^4^ Big Data Research Office, Chang Gung Memorial Hospital, Taoyuan, Taiwan; ^5^ Division of Rheumatology/Immunology/Allergy, Department of Internal Medicine, Tri-Service General Hospital, National Defense Medical Center, Taipei, Taiwan

**Keywords:** Sjögren syndrome, hydroxychloroquine, cancer, epidemiology, pharmacoepidemiology

## Abstract

Hydroxychloroquine inhibits systemic inflammation and autophagy and may thus have antineoplastic effects [1]. We investigated the effect of hydroxychloroquine on cancer risk in patients with primary Sjögren syndrome(pSS). We used the Taiwan National Health Insurance Database to compare cancer incidence between incident pSS patients with or without at least 6-month hydroxychloroquine use within a 1- or 3-year period. Propensity score matched landmark analysis was used. We included 4194 alive patients without cancer 1 year after pSS diagnosis from 2000 through 2005. The propensity score matched 1148 patients with at least 6-month hydroxychloroquine exposure at 1 year after diagnosis and 1148 patients without. Median follow-up after the 1-year landmark was 6 years. During follow up 62 hydroxychloroquine users and 56 non-hydroxychloroquine users developed cancer. Kaplan–Meier estimates showed no difference in overall survival between hydroxychloroquine users and non-users in the 1-year. Hydroxychloroquine was associated with a hazard ratio (HR) of 1.11 (95% CI, 0.78–1.60) in 1-year landmark analysis. In 3-year landmark analysis, hydroxychloroquine was associated with a HR for cancer of 1.37 (95% CI, 0.97–1.94). This propensity score matched landmark analysis of Taiwanese patients with incident pSS found that hydroxychloroquine was not associated with cancer risk nor protection.

## INTRODUCTION

Primary Sjögren syndrome (pSS) is a common autoimmune disease. The principle manifestations are dry mouth, dry eyes, swollen salivary gland, arthritis and skin lesions. Other systemic features are also reported such as the involvement of the lungs and peripheral nervous system [2]. In addition, pSS is associated with an increased risk for cancer. A recent meta-analysis summarized data from 14 studies found that patients with pSS had a pooled relative risk of 1.53 for overall cancer and 13.76 for non-Hodgkin lymphoma [3].

Hydroxychloroquine is a disease-modifying anti-rheumatic drug (DMARD) for pSS. The safety profiles of chloroquine and hydroxychloroquine are well-documented from the experiences of their use in treating malaria [4]. Inhibition of systemic inflammation and autophagy by hydroxychloroquine has been linked to antineoplastic effects. Currently, multiple randomized controlled trials have been conducted using hydroxychloroquine as an anticancer drug or as an add-on to existing chemotherapeutic regimens [1]. Several clinical trials in last 2 years with autophagy inhibition for the treatment of multiple neoplasms. Studies in melanoma, glioblastoma, pancreatic, breast, lung, and prostate cancer are testing chloroquine/hydroxychloroquine as a single agent or combination therapy [5]. However, the evidence of therapeutic benefits of hydroxychloroquine on cancer is controversial.

An interesting idea is to use hydroxychloroquine for secondary prevention of cancer in high risk population, for example, patients with pSS, given its antineoplastic effects and good safety profile in therapeutic doses. In this study, we used a large cohort of pSS from Taiwan National Health Insurance Database to test the hypothesis that hydroxychloroquine use for at least 6 months has the potential protective effect on cancer occurrence. To keep immortal time bias and confounding by indication to a minimum, we utilized the landmark analysis design [6]—which arbitrarily defines a fixed observation period from cohort entry to treatment assignment—to control immortal time bias, and propensity score [7] to balance the probability of receiving a specific treatment at the time of assignment. These methods ensure a fair comparison between hydroxychloroquine users and non-users for cancer risks.

## RESULTS

### Study population

**Figure 1:**
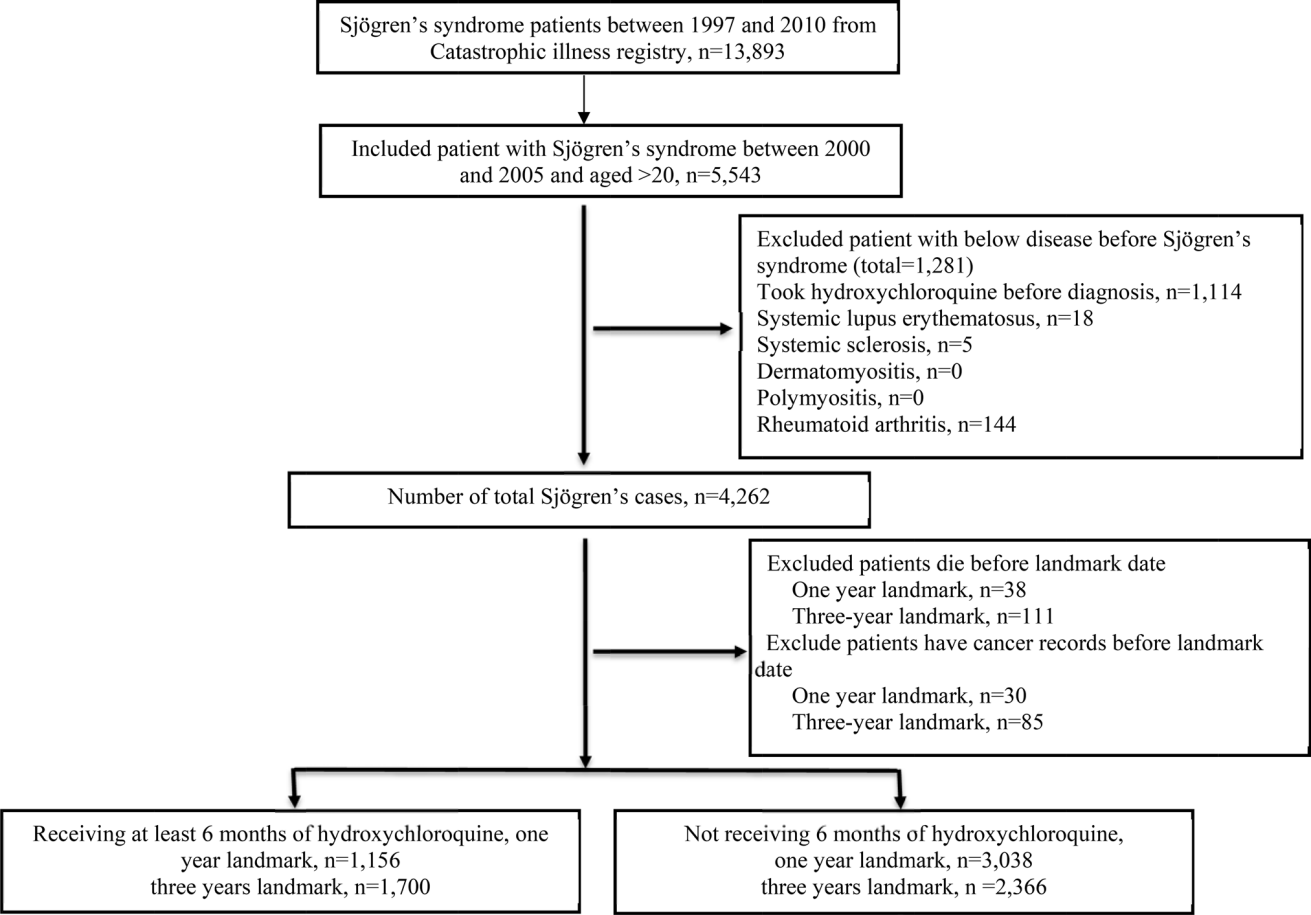
Flowchart of recruitment of subjects with Sjögren's syndrome from National Health Insurance Research Database between 2000 to 2005 in Taiwan

We identified 13,893 pSS patients who were older than 20 years from catastrophic illness registry. Between January 2000 and December 2005, we identified 5543 pSS patients of whom. 1281 patients were excluded because they had previous pSS diagnosis, received hydroxychloroquine before diagnosis or had secondary pSS with other autoimmune disease. A total 4262 patients with incident pSS (women: 3689 [86.56%]). Among them, 68 patients were excluded from the 1-year landmark analysis and 196 patients were excluded from the 3-year landmark analysis because of death and cancer diagnosis before landmark date (Figure 1). Table 1 shows the baseline characteristics of patients included in and excluded from the 1- and 3-year landmark analyses.

**Table 1:** Baseline characteristics of Sjögren’s syndrome cohort

	Entire cohort (*n* = 4,262)	One-year Landmark cohort			Three-year landmark cohort		
		Included patients (*n* = 4,194)	Excluded patients (*n* = 68)	*P* value	Included patients (*n* = 4,066)	Excluded patients (*n* = 196)	*P* value
**Age (years)**							
Median (interquartile range)	53 (44–63)	53 (44–63)	62 (51–72)	<.0001^a^	52 (43–63)	66 (53–74)	<.0001^a^
**Gender, No. (%)**							
Men	573 (13.44)	556 (13.26)	17 (25.00)	0.0049	524 (12.89)	49 (25.00)	<.0001
Women	3689 (86.56)	3638 (86.74)	51 (75.00)		3542 (87.11)	147 (75.00)	
**Place of residence, No. (%)**							
Urban	1388 (32.57)	1368 (32.62)	20 (29.41)	0.5578	1322 (32.51)	66 (33.67)	0.8337
Suburban	1295 (30.38)	1273 (30.35)	22 (32.35)		1239 (30.47)	56 (28.57)	
Rural	1484 (34.82)	1458 (34.76)	26 (38.24)		1413 (34.75)	71 (36.22)	
Unknown	95 (2.23)	95 (2.27)	--		92 (2.26)	3 (1.53)	
**Income level, No. (%)**							
Quintile 1	849 (19.92)	832 (19.84)	17 (25.00)	0.2303	800 (19.68)	49 (25.00)	0.0553
Quintile 2	453 (10.63)	449 (10.71)	4 (5.88)		436 (10.72)	17 (8.67)	
Quintile 2	1024 (24.03)	1002 (23.89)	22 (32.35)		967 (23.78)	57 (29.08)	
Quintile 4	738 (18.37)	775 (18.48)	8 (11.76)		759 (18.67)	24 (12.24)	
Quintile 5	1100 (25.81)	1083 (25.82)	17 (25.00)		1052 (25.87)	48 (24.49)	
Unknown	53 (1.24)	53 (1.26)	--		52 (1.28)	1 (0.51)	
**Occupation, No. (%)**							
Dependents of the insured individuals	1025 (24.05)	1005 (23.96)	20 (29.41)	0.0914	959 (23.59)	66 (33.67)	<.0001
Civil servants, teachers, military personnel, and veterans	397 (9.31)	395 (9.42)	2 (2.94)		390 (9.59)	7 (3.57)	
Nonmanual workers and professionals	1000 (23.46)	990 (23.61)	10 (14.71)		976 (24.00)	24 (12.24)	
Manual workers	1401 (32.87)	1375 (32.78)	26 (38.24)		1332 (32.76)	69 (35.20)	
Other	439 (10.30)	429 (10.23)	10 (14.71)		409 (10.06)	30 (15.31)	
**Charlson comorbidity index, No. (%)**							
1–2	3221 (75.57)	3187 (75.99)	34 (50.00)	<.0001	3111 (76.51)	110 (56.12)	<.0001
3–4	533 (12.51)	517 (12.33)	16 (12.53)		497 (12.22)	36 (18.37)	
≥ 4	508 (11.92)	490 (11.68)	18 (26.47)		458 (11.26)	50 (25.51)	
**Medications, No. (%)**							
Azathioprine	29 (0.68)	28 (0.67)	1 (1.47)	0.3737^b^	27 (0.66)	2 (1.02)	0.3881^b^
Prednisolone	1993 (46.76)	1956 (46.64)	37 (54.41)	0.2025	1901 (46.75)	92 (46.94)	0.9595
Methotrexate	35 (0.82)	35 (0.83)	--	1.0000^b^	34 (0.84)	1 (0.51)	1.0000^b^
Cyclosporin	11 (0.26)	10 (0.24)	1 (1.47)	0.1623^b^	9 (0.22)	2 (1.02)	0.0881^b^

^a^Wilcoxon rank sum test.

^b^Fisher’s Exact Test.

### Matching

In the 1-year landmark analysis, we included 4194 patients who were alive 1 year after an initial diagnosis of pSS. Among them, 1156 patients had a duration of hydroxychloroquine exposure of at least 6 months. Variables included in the propensity score calculation did not significantly differ between hydroxychloroquine users and non-users after matching, which confirms the success of matching (Table 2). In the 3-year landmark analysis, we included 4066 patients who were alive 3 years after an initial diagnosis of pSS. There were 1700 hydroxychloroquine users at the 3-year landmark. After matching, there were no significant differences in variables for propensity scores (Table 3).

**Table 2:** Comparison of patients exposed to or unexposed to hydroxychloroquine within one year from initial diagnosis of Sjögren’s syndrome before and after matching

	Exposure groups before matching			Exposure groups after matching		
	hydroxychloroquine users (*n* = 1,156)	hydroxychloroquine nonusers (*n* = 3,038)	Standardized difference	hydroxychloroquine users (*n* = 1,148)	hydroxychloroquine nonusers (*n* = 1,148)	Standardized difference
**Age (years)**						
Median (interquartile range)	52 (43–62)	53 (44–64)	–0.105	52 (43–62)	51 (43–62)	–0.001
**Gender, No. (%)**						
Men	140 (12.11)	416 (13.69)	–0.105	139 (12.11)	143 (12.46)	–0.001
Women	1016 (87.89)	2622 (86.31)		1009 (87.89)	1005 (87.54)	
**Place of residence, No. (%)**						
Urban	355 (30.71)	1013 (33.34)	0.0748	354 (30.84)	353 (30.75)	0.0140
Suburban	364 (31.49)	909 (29.92)		360 (31.36)	367 (31.97)	
Rural	416 (35.99)	1042 (34.30)		413 (35.98)	407 (35.45)	
Unknown	21 (1.82)	74 (2.44)		21 (1.83)	21 (1.83)	
**Income level, No. (%)**						
Quintile 1	201 (17.39)	631 (20.77)	0.1200	201 (17.51)	208 (18.12)	0.0442
Quintile 2	129 (11.16)	320 (10.53)		128 (11.15)	127 (11.06)	
Quintile 2	269 (23.27)	733 (24.13)		269 (23.43)	278 (24.22)	
Quintile 4	238 (20.59)	537 (17.68)		233 (20.30)	221 (19.25)	
Quintile 5	309 (26.73)	774 (25.48)		307 (26.74)	307 (26.74)	
Unknown	10 (0.87)	43 (1.42)		10 (0.87)	7 (0.61)	
**Occupation, No. (%)**						
Dependents of the insured individuals	266 (23.01)	739 (24.33)	0.1213	265 (23.08)	261 (22.74)	0.0289
Civil servants, teachers, military personnel, and veterans	115 (9.95)	280 (9.22)		115 (10.02)	119 (10.37)	
Nonmanual workers and professionals	294 (25.43)	696 (22.91)		292 (25.44)	289 (25.17)	
Manual workers	389 (33.65)	986 (32.46)		384 (33.45)	379 (33.01)	
Other	92 (7.96)	337 (11.09)		92 (8.01)	100 (8.71)	
**Charlson comorbidity index, No. (%)**						
1–2	890 (76.99)	2297 (75.61)	0.1030	890 (77.53)	895 (77.96)	0.1267
3–4	140 (12.11)	377 (12.41)		138 (12.02)	120 (10.45)	
≥ 4	126 (10.90)	364 (11.98)		120 (10.45)	133 (11.59)	
**Medications, No. (%)**						
Azathioprine	5 (0.43)	23 (0.76)	–0.0420	5 (0.44)	5 (0.44)	0.0000
Prednisolone	537 (46.45)	1419 (46.71)	–0.0050	532 (46.34)	528 (45.99)	0.0070
Methotrexate	9 (0.78)	26 (0.86)	–0.0090	9 (0.78)	7 (0.61)	0.0209
Cyclosporin	4 (0.35)	6 (0.20)	0.0285	3 (0.26)	4 (0.35)	–0.0160

**Table 3:** Comparison of patients exposed to or unexposed to hydroxychloroquine within three years from initial diagnosis of Sjögren’s syndrome before and after matching

	Exposure groups before matching			Exposure groups after matching		
	hydroxychloroquine users (*n* = 1,700)	hydroxychloroquine nonusers (*n* = 2,366)	Standardized difference	hydroxychloroquine users (*n* = 1,682)	hydroxychloroquine nonusers (*n* = 1,682)	Standardized difference
**Age (years)**						
Median (interquartile range)	52 (42–61)	53 (44–64)	–0.0960	52 (43–61)	51 (43–61)	0.0192
**Gender, No. (%)**						
Men	190 (11.18)	334 (14.12)	–0.0960	189 (11.24)	189 (11.24)	0.0192
Women	1510 (88.82)	2032 (85.88)		1493 (88.76)	1493 (88.76)	
**Place of residence, No. (%)**						
Urban	543 (31.94)	779 (32.92)	0.0759	538 (31.99)	556 (33.06)	0.0693
Suburban	530 (31.18)	709 (29.97)		525 (31.21)	509 (30.26)	
Rural	594 (34.94)	819 (34.62)		586 (34.84)	581 (34.54)	
Unknown	33 (1.94)	59 (2.49)		33 (1.96)	36 (2.14)	
**Income level, No. (%)**						
Quintile 1	300 (17.65)	500 (21.13)	0.1185	299 (17.78)	307 (18.25)	0.0796
Quintile 2	190 (11.18)	246 (10.40)		187 (11.12)	199 (11.83)	
Quintile 2	375 (22.06)	592 (25.02)		375 (22.29)	377 (22.41)	
Quintile 4	348 (20.47)	411 (17.37)		341 (20.27)	326 (19.38)	
Quintile 5	467 (27.47)	585 (24.73)		460 (27.35)	448 (26.63)	
Unknown	20 (1.18)	32 (1.35)		20 (1.19)	25 (1.49)	
**Occupation, No. (%)**						
Dependents of the insured individuals	387 (22.76)	572 (24.18)	0.1168	386 (22.95)	375 (22.29)	0.0451
Civil servants, teachers, military personnel, and veterans	176 (10.35)	214 (9.04)		174 (10.34)	158 (9.39)	
Nonmanual workers and professionals	440 (25.88)	536 (22.65)		431 (25.62)	445 (26.46)	
Manual workers	553 (32.53)	779 (32.92)		548 (32.58)	557 (33.12)	
Other	144 (8.47)	265 (11.20)		143 (8.50)	147 (8.74)	
**Charlson comorbidity index, No. (%)**						
1–2	1303 (76.65)	1808 (76.42)	0.1017	1300 (77.29)	1316 (78.24)	0.0970
3–4	210 (12.35)	287 (12.13)		206 (12.25)	198 (11.77)	
≥ 4	187 (11.00)	271 (11.45)		176 (10.46)	168 (9.99)	
**Medications, No. (%)**						
Azathioprine	10 (0.59)	17 (0.72)	-0.0390	10 (0.59)	11 (0.65)	–0.034
Prednisolone	792 (46.59)	1109 (46.87)	-0.0050	786(46.73)	808 (48.04)	–0.020
Methotrexate	14 (0.82)	20 (0.85)	-0.0060	14 (0.83)	14 (0.83)	–0.004
Cyclosporin	3 (0.18)	6 (0.25)	0.0129	3 (0.18)	3 (0.18)	0.0304

### Outcomes after matching

**Figure 2:**
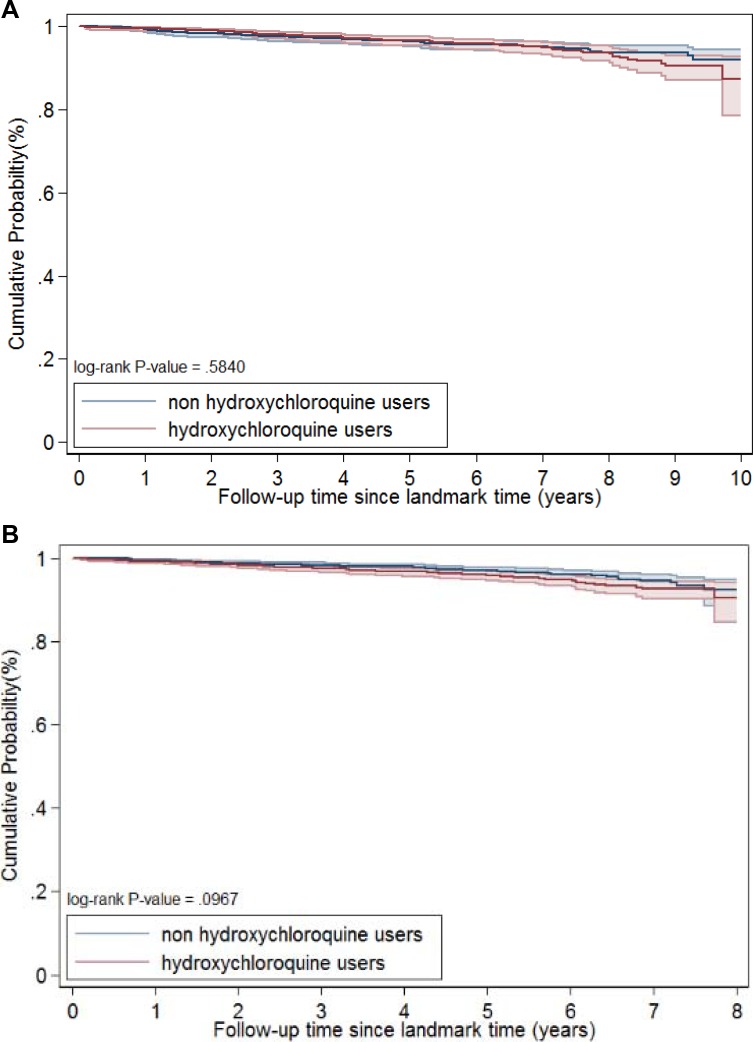
Survival curve after propensity score matching, one-year landmark (**A**), and three-years landmark (**B**).

Median follow-up was 6 and 4 years for the 1- and 3-year landmark analyses, respectively. There was no significant difference in cancer incidence between hydroxychloroquine users and non-users in the 1- or 3-year landmark analyses (Table 4). Kaplan–Meier analysis showed no difference in survival between hydroxychloroquine users and non-users in the 1-year (log-rank test, *p* = 0.58) or 3-year (log-rank test, *p* = 0.10) landmark analyses (Figure 2). The HRs (95% CI) were 1.11 (0.78–1.60) for the 1-year landmark analysis and 1.37 (0.97–1.94) for the 3-year landmark analysis. Analysis of lymphoma incidence showed that the HR (95% CI) was 1.00 (0.29–3.45) in the 1-year landmark analysis and 3.12 (0.63–15.54) in the 3-year landmark analysis.

**Table 4:** Comparison the morbidity rate of patients exposed to or unexposed to hydroxychloroquine after matching

	One-year landmark analysis			Three-year landmark cohort		
	hydroxychloroquine users (*n* = 1,148)	hydroxychloroquine nonusers (*n* = 1,148)	*P* value	Hydroxychloroquine users (*n* = 1,682)	hydroxychloroquine nonusers (*n* = 1,682)	*P* value
Median (interquartile range)*	6.38 (4.79–8.07)	6.49 (4.91–7.95)	0.7528 ^a^	4.40 (2.93–6.02)	4.71 (3.16–6.25)	0.0007^a^
Cancer	62 (5.40)	56 (4.88)	0.3931	71 (4.22)	56 (3.33)	0.1748
**Morbidity (%)**						
One year from landmark	5 (0.44)	7 (0.61)	0.5190	13 (0.77)	10 (0.59)	0.9913
Two years from landmark	11 (0.96)	18 (1.57)		29 (1.72)	21 (1.25)	
Five years from landmark	36 (3.14)	39 (3.40)		56 (3.33)	41 (2.44)	
Ten years from landmark	62 (5.40)	56 (4.88)		71 (4.22)	56 (3.33)	

*The follow-up years since the time point of landmark.

^a^Wilcoxon rank sum test.

### Propensity score adjustment analysis

When we used propensity score adjustment for all patients included in the 1- and 3-year landmark analyses, the results were similar to those obtained in our main analysis. In the 1-year landmark analysis, hydroxychloroquine was associated with an unadjusted HR (95% CI) of 1.10 (0.82–1.47) and a propensity score adjusted HR of 1.17 (0.87–1.57). In the 3-year analysis, the unadjusted and propensity score adjusted HRs were 1.15 (0.85–1.58) and 1.28 (0.94–1.75), respectively.

The results for lymphoma were not statistically significant. In the 1-year landmark analysis, hydroxychloroquine was associated with an unadjusted HR (95% CI) of 1.22 (0.43–3.50) and a propensity score adjusted HR of 1.26 (0.44–3.62). In the 3-year analysis, the unadjusted and propensity score adjusted HRs were 1.77 (0.55–5.72) and 1.88 (0.56–6.35), respectively.

## DISCUSSION

This is the first population-based study to investigate the effects of hydroxychloroquine on malignancy in pSS patients. Our evidence suggests that hydroxychloroquine use is not associated with cancer risk in patients with pSS. The absence of association with cancer in hydroxychloroquine users persisted after adjustment for cancer risk factors and covariates including age, sex, socioeconomic factors, medication history, and comorbidities. In addition, hydroxychloroquine was not associated with any protective effect on the risk of lymphoma.

Cancer risk is significantly higher in patients with pSS than controls in a previous study. The risk was highest for non-Hodgkin lymphoma (pooled relative risk, 13.76; 95% CI, 8.53–18.99)[3]. Our recent nationwide population cohort study in Taiwan showed that cancer incidence increased in patients with pSS compared to general population. The standardized incidence ratio for all cancer was significantly higher among patients with pSS (1.19; 95% CI, 1.08–1.30) [8]. Since hydroxychloroquine has been found to have anti-cancer effects and its safety profile is well-known, it is important to determine if hydroxychloroquine is effective in preventing cancer in this patient subgroup [9].

Recent research suggests that this antimalarial drug and its related derivatives are potential anti-cancer agents [10]. The antineoplastic activity of hydroxychloroquine probably stems from its effect on the autophagy inhibition, which has long been observed as a potentially effective mechanism to improve therapeutic profile of anti-cancer medications. For example, a recent study found that hydroxychloroquine may restore antiestrogen sensitivity of estrogenic receptor positive breast cancer [11]. However, multiple clinical trials to examine the antineoplastic activity of hydroxychloroquine or chloroquine, its parent compound, fail to demonstrate beneficial effects of both drugs to act as stand-alone anticancer intervention [12]. Ongoing clinical trials studying the roles of hydroxychloroquine, chloroquine or their derivatives as stand-alone regimen or as an adjuvant to boost other anticancer regimens are under investigation. However, accumulative evidence so far does not support hydroxychloroquine's autophagy inhibition effect as a mean to improve therapeutic profile of current regimens.

In contrast to previous evidence, which mainly use hydroxychloroquine as a direct anti-cancer agent, our study used real-world data to understand whether hydroxychloroquine has any effect on primary prevention of cancer. We hypothesized that hydroxychloroquine use in patients with pSS, who are already at a higher risk for cancer especially lymphoma, would retard their cancer risk. However, observational studies are subject to several biases that can obscure treatment effects. Immortal time bias and confounding by indication are two well-known biases and are difficult to address. Immortal time bias generally causes spurious inflation of a beneficial treatment effect because of the guaranteed period of survival in the treatment group, which is present by design. Conversely, confounding by indication generally favors the unexposed group because treated patients tend to have poorer outcomes. We therefore use a sophisticated study design, landmark analysis plus propensity score, to suppress both biases to a minimum. Our data did not support hydroxychloroquine use at usual dose for at least half year has any effect on cancer risk. However, hydroxychloroquine did not increase the cancer risk either. Therefore, hydroxychloroquine can be used as a DMARD for pSS or other rheumatic diseases without consideration of its effect on autophagy inhibition that would influence cancer risk.

Several possible reasons may account for our neutral findings. Firstly, hydroxychloroquine used as an DMARD rarely exceed 400 mg/day, while previous studies targeting on anti-cancer activity generally evaluate higher doses. For example, a recent pilot study recommended the use hydroxychloroquine at a dose of 1000 mg/day in combination with erlotinib for a phase II study focusing on the treatment of non-small-cell lung cancer [13]. In a dose of usual care, the anticancer effect of hydroxychloroquine is questionable. Secondly, patients with rheumatic diseases, including pSS, are already at a high risk of cancers [8]. The mechanisms of an increased cancer risk in pSS are generally thought to involve a failure of immunosurveillance on cancer, which is not related to treatment effect of hydroxychloroquine. Therefore, even hydroxychloroquine has an antineoplastic effect, it is probably too small to overcome existing risks in pSS patients. In this regard, the decision to prescribe hydroxychloroquine should focus on its effects on rheumatic diseases, rather than on the hypothetical anti-cancer activity.

Treatment of systemic symptoms of pSS has been conducted in multiple trials, but none of the investigational therapies have been approved till now [14]. Medications such as cyclophosphamide, azathioprine, or mycophenolate mofetil may be prescribed by clinicians for vasculitis related to pSS. Cyclophosphamide and azathioprine for treating other autoimmune disease has bene found to increase cancer risks in previous studies. [15, 16]. However, mycophenolate mofetil inhibits tumor growth and angiogenesis in previous report [17]. Further research is required for more evidence.

This study has some potential limitations and several possibilities to explain why hydroxychloroquine could not reduce cancer risk in pSS patients. Firstly, because we used landmarks of 1 and 3 years our result showing that hydroxychloroquine has no effect on cancer risk only applies to patients who are alive at these time points. Although our methods address the problem of immortal time bias, selection bias is still a possibility. However, the landmark analyses showed similar trends and we thus have no reason to believe that landmark selection interacted with outcomes. Furthermore, we use propensity score adjustment to further verify our results and reached similar conclusion. Secondly, the hydroxychloroquine dosage used to treat pSS patients is usually less than 400 mg per day, the dosage required for hydroxychloroquine to be antineoplastic is high up to 1000 mg, a dosage that can never be prescribed to treat pSS patients [13]. Whether a higher hydroxychloroquine dose would have any beneficial effect on cancer incidence remains to be elucidated. Thirdly, in considering the inevitable side effects like hyperpigmentation (a major concern in oriental population), the poor compliance for some pSS patients may also account for the inadequacy of hydroxychloroquine to be cancer-preventive. Finally, this study used propensity to handle confounding by indication bias. There may be residual confounders that have not been considered in our study. A proper method to contain residual confounding includes propensity score calibration which used external data to adjust effect estimates for unmeasured confounders. However, we used NHI data from the entire population, which linkable identification is encrypted to prevent confidentiality break. Therefore, we are not able to conduct propensity score calibration. We did multiple sensitivity analysis, including analyses for 2 different landmarks and the use of both propensity score matching and adjustment. All of which show similar results. In addition, our data matched the results of previous early clinical trials. Nevertheless, further study is needed to confirm our results.

In conclusion, this propensity score matched landmark analysis of Taiwanese patients with incident pSS showed that hydroxychloroquine had a neutral effect on cancer risk. Future studies should examine if a higher hydroxychloroquine dose or a longer exposure would modify cancer risk in patients with pSS.

## MATERIALS AND METHODS

Ethics approval was obtained from the Institutional Review Board of Chang Gung Memorial Hospital. Patient consent was deemed unnecessary because confidential data were totally anonymized. This propensity score matched landmark study compared cancer incidence in pSS patients who were and were not treated with hydroxychloroquine for at least 6 months.

### Data source

Our primary data source was the National Health Insurance (NHI) system of Taiwan, which was established in 1995. The NHI is a single-payer system and all citizens are required by law to enroll. Coverage is therefore exceptionally high—99.5% in 2010 [18]. The NHI Database contains detailed information on registration and original claims data, including demographic data, dates of visits, diagnostic codes and prescriptions, gender, date of birth, place of residence, insurance details, family relationships, vital status, and clinical information including dates of inpatient and outpatient visits, medical diagnoses, medical expenditures, prescription details, vaccination status, examinations, and operations and procedures. A unique personal identifier assigned to each resident of Taiwan allows all information in the database to be linkable internally and externally to other government held data. To ensure confidentiality, unique personal identifiers are encrypted before data are released to researchers, but the identifier remains unique for each beneficiary in the database, to facilitate internal linkage of records. The diagnostic coding system in the NHI database follows the International Classification of Diseases, Ninth Revision, Clinical Modification (ICD-9-CM). The validity, representativeness, and clinical consistency of the NHI database have been described elsewhere [19].

In summary, NHI in Taiwan covers more than 99% of Taiwanese citizens and gathers routine medical service information. This database records important usage of health services and provides resource for researchers to investigate the longitudinal or cross-sectional association between potential risk factors and disease outcomes base on possible research questions. However, the main object of NHI is designed for the usage of daily health service in Taiwan. Clinical diagnoses and medical prescriptions might vary across different physicians and specialist. This database also lacks of detailed medical information such as laboratory results.

### Case definition of primary Sjögren syndrome and cancer

We identified patients with pSS by analyzing the “catastrophic” illness registry for the period from 2000 through 2005. This registry also uses ICD-9-CM codes. If patients in Taiwan have a major disease, including autoimmune disease such as pSS and any cancer, they are eligible for a “catastrophic” illness certificate that waives outpatient and inpatient co-payments. Issuance of this certificate requires the filing of an application by physicians, along with information sufficient to classify autoimmune diseases or all evidence supporting the cancer diagnosis, including findings from cytology, pathology, and clinical studies, laboratory testing, and imaging studies. The certificate is issued after a formal review by expert panels commissioned by the NHI Administration. Both the preliminary European classification criteria for Sjögren's syndrome [20] and classification criteria by the American-European Consensus Group [21] were used by review panel to determine the validity of pSS. We identified patients with pSS by using ICD-9-CM code 710.2, and only incident cases between 2000 to 2005 were included in this study. We excluded patients with systemic lupus erythematosus, systemic sclerosis, dermatomyositis, polymyositis, or rheumatoid arthritis and those who had used hydroxychloroquine before the first date of pSS diagnosis.

### Treatment assignment

Exposure to hydroxychloroquine was defined as receipt of a hydroxychloroquine prescription for 6 months or longer. Patients may receive hydroxychloroquine after a pSS diagnosis; however, prescription of hydroxychloroquine often lags the initial diagnosis of pSS. Therefore, the date on which 6 months of hydroxychloroquine therapy is completed is likely to vary considerably from person to person. Hydroxychloroquine treatment dosage for pSS is usually less than 400 mg per day in Taiwan. In this study, we used landmark analysis to examine the effect of hydroxychloroquine exposure on cancer [6]. Specifically, we selected a fixed time after pSS diagnosis (1 year and 3 years) a priori at which time point patient follow-up started (index date). Only patients alive and free of the major outcome at the landmark date were included in the analysis. Treatment assignment for hydroxychloroquine was based on exposure before the landmark date. Hydroxychloroquine exposure was only evaluated between pSS diagnosis and the index date (the exposure window), and outcome was evaluated from index date. Patients with outcomes occurring during exposure were excluded from subsequent analysis, to avoid immortal time bias [22].

We classified patients by exposure to hydroxychloroquine for at least 6 months during a 1-year period. To ensure that every patient had equal exposure window for hydroxychloroquine exposure and to avoid immortal time bias, patients who had cancer or died before the index date were excluded from the analysis. In addition, we used an exposure window of 3 years in a sensitivity analysis to see whether the arbitrarily chosen landmark would affect the results. Patients were followed up until the date of death, cancer development, or 31 December 2010, whichever occurred first.

### Covariates

Covariates included patient characteristics (age, sex), socioeconomic factors, comorbidities, and co-medication. We considered these factors pertinent to the physicians’ prescribing decisions. Only records for the 5-year period before the initial diagnosis of pSS were used to evaluate comorbidities and drug treatment. The Dartmouth–Manitoba version of the Charlson Comorbidity Index was used to defined comorbidities. Details of the ICD-9-CM codes for these comorbidities are shown in Supplementary Table 1. Co-medications included azathioprine, prednisolone, methotrexate, cyclosporine, and any combination of these medications [23, 24].

Individual place of residence was defined as one of the 369 towns or districts in Taiwan. Level of urbanization was designated as urban, suburban, or rural [25]. Occupation was classified into (1) civil servants, teachers, military personnel, and veterans, (2) non-manual workers and professionals, (3) manual workers, (4) other, and (5) dependents. Income level was approximated by using the salaries of employees and civil servants and the business income of employers.

### Outcomes

The catastrophic illness registry was used to identify cancer cases (ICD-9-CM codes 140–208) and date of cancer diagnosis after the landmark date. To ascertain the validity of cancer diagnoses in the NHI database, we linked the NHI with the National Cancer Registry, which served as the reference standard. Between 2001 and 2012, we identified 835,967 patients with cancer from the NHI database, 855,794 from the National Cancer Registry. The positive and predictive values of NHI database cancer diagnosis were 94% and 99% for all cancers, respectively.

### Statistical analysis

We examined the characteristics of the pSS cohort. Categorical variables are presented as percentages, and continuous variables are expressed as median (interquartile range), as appropriate. To compare baseline characteristics, we used the Wilcoxon rank sum test for continuous variables and the Pearson χ2 test or Fisher exact test for categorical variables. In a landmark analysis, a period of time between a baseline date (cohort entry) and a study start date (the landmark date) is designated the exposure period and chosen a priori. All exposures are classified during this time period; only outcomes that occur after the landmark date are counted in the analysis. Participants who experience the outcome of interest (cancer and lymphoma) during the exposure window determined by 1- or 3-year landmark date are excluded from analyses to avoid reverse causality and immortal time bias (which would tend to overestimate the benefit of the exposure). Exposures that occur after the landmark date do not affect group assignment [22, 26].

We used a propensity score matched landmark design to investigate the effect of cancer morbidity among hydroxychloroquine users. Propensity score was calculated by logistic regression models, which indicates the conditional probability of receiving hydroxychloroquine and was adjusted by age, sex, socioeconomic factors, medications, and comorbidities. Then we matched hydroxychloroquine-exposed patients to non-exposed patients at a ratio of 1 to 1 on the basis of the logit of the propensity score, using calipers with a width equal to 0.2 of the standard deviation of the logit of the propensity score [27]. In a sensitivity analysis we also used propensity score adjustment regression for the entire included cohort, to compare morbidity risks between hydroxychloroquine users and non-users [28]. The hazard ratio (HR) for cancer occurrence was determined with Cox proportional hazards analysis. Lymphoma was used as another outcome in sensitivity analysis. Kaplan–Meier plots were used to estimate cumulative probability, and the log-rank test used to assess differences between groups. Analyses were performed with SAS version 9.4. Differences were considered statistically significant when the 95% confidence interval (CI) did not include unity or when P was < 0.05. All tests were two-sided.

## SUPPLEMENTARY MATERIALS TABLE



## Abbreviations

pSS: primary Sjögren syndrome
DMARD: disease-modifying anti-rheumatic drug
NHI: National Health Insurance
HR: hazard ratio
CI: confidence interval
