# Supplementary material for: Hydroxychloroquine and risk of cancer in patients with primary Sjögren syndrome: propensity score matched landmark analysis
Source: Oncotarget. 2017 Jul 6;8(46):80461–71. doi: 10.18632/oncotarget.19057 (PMC5655212; doi:10.18632/oncotarget.19057)
Supplement: Supplementary file 1 [file oncotarget-08-80461-s001.pdf]

# Hydroxychloroquine and risk of cancer in patients with primary Sjögren syndrome: propensity score matched landmark analysis

## Supplementary Materials

### Supplementary Table 1: Dartmouth-manitoba's charlson comorbidity index codes

| Comorbidity | Weight | ICD-9-CM diagnosis codes | ICD-9-CM procedure codes |
|---|---|---|---|
| Myocardial infarct | 1 | 410.xx, 412 | |
| Congestive heart failure | 1 | 402.01, 402.11, 402.91, 425.x, 428.x, 429.3, 404.01, 404.03, 404.11, 404.13, 404.91, 404.93 | |
| Peripheral vascular disease | 1 | 440.x, 441.x, 442.x, 443.1-443.9, 447.1, 785.4 | 38.13-38.14, 38.16, 38.18, 38.33-38.34, 38.36, 38.38, 38.43-38.44, 38.46, 38.48, 39.22-39.26, 39.29 |
| Cerebrovascular disease | 1 | 362.34, 430-436, 437-437.1, 437.9, 438, 781.4, 784.3,997.0 | 38.12, 38.42 |
| Dementia | 1 | 290.x, 331-331.2 | |
| Chronic pulmonary disease | 1 | 415.0, 416.8-416.9, 491.x-494, 496 | |
| Rheumatologic disease | 1 | 710.x, 714.x | |
| Peptic ulcer disease | 1 | 531.xx-534.xx | |
| Mild liver disease | 1 | 571.2, 571.5-571.6, 571.8-571. 9 | |
| Diabetes | 1 | 250.0x-250.3x | |
| Diabetes with end organ damage | 2 | 250.4x-250.9x | |
| Hemiplegia or paraplegia | 2 | 342.x, 344.x | |
| Renal disease | 2 | 585-586, V42.0, V45.1, V56.x, | 39.27, 39.42, 39.93-39.95, 54.98 |
| Any malignancy, include leukemia and lymphoma | 2 | 140.x-171.x, 174.x-195.x, 200.xx-208.x, 273.0, 273.3, V10.46, | 60.5, 62.4-62.41 |
| Moderate or severe liver disease | 3 | 572.2-572.4, 456.0-456.2x | 39.1, 42.91 |
| Metastatic solid tumor | 6 | 196.x-199.x | |
| AIDS | 6 | 042.x-044.x | |
